# Distribution of Isolated Pathogens and Resistance Patterns in Non-Ventilator Hospital-Acquired Pneumonia at King Hamad University Hospital (KHUH), Bahrain: A Retrospective Study

**DOI:** 10.7759/cureus.101482

**Published:** 2026-01-13

**Authors:** Layal E Omaruddin, Ayat A Ziedan, Osama Zeidan, Omaima A Shaaban, Anas A Zeidan, Duaa Behbehani, Nadine Zankar

**Affiliations:** 1 Internal Medicine, Royal College of Surgeons in Ireland - Bahrain, Busaiteen, BHR; 2 Internal Medicine, Sultan Qaboos University, Muscat, OMN; 3 Medicine, Royal College of Surgeons in Ireland - Bahrain, Busaiteen, BHR; 4 Pediatrics, Royal College of Surgeons in Ireland - Bahrain, Busaiteen, BHR; 5 Internal Medicine, East Lancashire Hospitals NHS Trust, Blackburn, GBR; 6 Pulmonology, Gulf Gate Medical Complex, Manama, BHR

**Keywords:** antimicrobial resistance, bahrain, empirical antibiotic therapy, hospital-acquired pneumonia, klebsiella pneumoniae, non-ventilator-associated pneumonia

## Abstract

Background

Non-ventilator hospital-acquired pneumonia (NV-HAP), defined as pneumonia developing ≥48 hours after admission in non-intubated patients, is increasingly recognized as a major contributor to hospital morbidity and mortality but remains under-studied compared with ventilator-associated pneumonia (VAP), particularly in the Gulf region. In Bahrain, published NV-HAP data are absent, limiting locally informed empiric therapy and antimicrobial stewardship. This study aimed to characterize bacterial pathogens causing culture-confirmed NV-HAP in adults at a tertiary center in Bahrain and to describe antimicrobial resistance patterns and temporal resistance trends.

Materials and methods

A retrospective cross-sectional study was conducted at King Hamad University Hospital (KHUH), Bahrain. Adult patients (≥18 years) with clinically and radiographically confirmed NV-HAP and positive sputum or bronchoalveolar lavage (BAL) cultures between January 2017 and March 2022 were included. NV-HAP required onset ≥48 hours after admission in non-intubated patients. Exclusion criteria included VAP, non-bacterial infections, prior antimicrobial therapy, restricted clinical trials, and immunocompromising conditions. ICD-10 code U69.01 was used for case identification, followed by manual chart review to confirm NV-HAP. Culture thresholds were >10⁴-10⁵ CFU/mL for sputum and >10³-10⁴ CFU/mL for BAL. Organism identification used MALDI-TOF, and susceptibility testing used the BD Phoenix M50 system. Multidrug resistance (MDR) was defined as non-susceptibility to ≥1 agent in ≥3 antimicrobial classes. Statistical analysis was performed using SPSS v25, with p ≤ 0.05 considered significant.

Results

Of 583 screened hospital-acquired pneumonia cases, 184 culture-confirmed NV-HAP cases met the inclusion criteria. Patients were predominantly male (65.2%) and elderly (≥75 years: 58.7%). Older patients had significantly higher comorbidity burdens (p < 0.01). Twenty bacterial species were identified; the most frequent pathogens were Klebsiella pneumoniae (38.59%), Pseudomonas aeruginosa (26.09%), Staphylococcus aureus (8.70%), Stenotrophomonas maltophilia (6.52%), and Acinetobacter baumannii (3.80%) (p = 0.014). K. pneumoniae was significantly more frequent in patients ≥ 75 years (44% vs 30%, p < 0.01). MDR was observed in 97.56% of K. pneumoniae isolates and was more common in older patients (p = 0.02). Resistance to ceftazidime, cefuroxime, levofloxacin, meropenem, and piperacillin-tazobactam increased significantly over time. P. aeruginosa showed expected intrinsic resistance patterns, with preserved susceptibility to aminoglycosides; MDR isolates comprised 14.6%.

Discussion

This first Bahrain NV-HAP study demonstrates a predominantly Gram-negative pathogen profile dominated by K. pneumoniae and P. aeruginosa, with low A. baumannii compared with regional VAP-focused studies, supporting distinct NV-HAP microbiology. The association between advanced age and K. pneumoniae, combined with high MDR rates and worsening resistance trends, has important implications for empiric therapy and antimicrobial stewardship.

Conclusion

NV-HAP in Bahrain is characterized by a high burden of MDR K. pneumoniae, particularly among elderly patients, and increasing resistance to commonly used agents. These findings highlight the need to revise empiric treatment strategies and strengthen antimicrobial stewardship, providing baseline data to guide NV-HAP management and regional surveillance.

## Introduction

Hospital-acquired pneumonia (HAP) is broadly categorized into ventilator-associated pneumonia (VAP) and non-ventilator hospital-acquired pneumonia (NV-HAP). NV-HAP is defined as pneumonia occurring ≥48 hours after admission in patients not receiving mechanical ventilation [1]. Despite this clear distinction, NV-HAP has historically received far less attention than VAP, even though evidence suggests it may represent the majority of HAP cases in acute care hospitals [2]. Concern regarding NV-HAP has grown in recent years: In 2020, US healthcare leaders established the National Organization to Prevent Hospital-Acquired Pneumonia (NOHAP) to highlight NV-HAP as one of the most common and morbid hospital-associated infections and to call for greater surveillance and prevention efforts [3]. Large contemporary surveillance data further reinforce its significance: An analysis of 284 US hospitals using standardized electronic criteria found that NV-HAP accounted for approximately 7% of all in-hospital deaths [4]. Additionally, NV-HAP is associated with substantially longer hospital stays and higher morbidity, underscoring the urgent need for focused research and prevention strategies [4].

Existing literature, especially from the Middle East, has largely concentrated on VAP in intensive care settings, highlighting a regional knowledge gap regarding NV-HAP [5]. For example, a decade-long surveillance study in Lebanon reported on VAP incidence and pathogens in a tertiary ICU [6], reflecting how Gulf and neighboring countries have dominated pneumonia research with VAP-focused studies (e.g., Kanafani et al., 2019) rather than NV-HAP [6]. In contrast, published investigations of NV-HAP in the Gulf are scarce or non-existent. In Bahrain, published data on NV-HAP are absent, as existing local work has focused primarily on VAP rather than NV-HAP [7]. This lack of NV-HAP-specific epidemiological data highlights the need for studies characterizing its burden and microbiological patterns in the Bahraini healthcare context. Unlike VAP (confined to mechanically ventilated patients), NV-HAP affects a broad hospital population, often older adults on general wards. Indeed, HAP disproportionately impacts elderly, frail patients, who frequently cannot tolerate invasive diagnostic techniques and are at higher risk due to factors like aspiration [8]. The current study thus addresses a critical gap by focusing on NV-HAP in an adult inpatient population (predominantly elderly) in Bahrain, a setting where data have been extremely limited.

Bahrain’s healthcare setting presents unique challenges and motivations for studying NV-HAP. The country’s population is demographically diverse and highly mobile, hosting a large expatriate community and significant travel, which may influence the spectrum of pathogens and antimicrobial resistance patterns in its hospitals [5]. Moreover, as in other Gulf nations, Bahrain faces a high burden of multidrug-resistant (MDR) organisms in hospital infections, driven by factors such as antibiotic overuse and continuous population movement [5]. Regional surveillance indicates that HAP/VAP cases in Gulf Cooperation Council hospitals are often caused by difficult-to-treat bacteria like Acinetobacter baumannii, Pseudomonas aeruginosa, and Klebsiella pneumoniae, with high rates of MDR reported among these pathogens [5]. These circumstances make empiric treatment of HAP especially challenging and reinforce the need for local data. In Bahrain, clinicians have lacked local evidence to guide therapy (“we did not have previous studies in our country to compare” [7], which this study aims to provide. By characterizing NV-HAP microbial epidemiology and resistance patterns in Bahrain, we can inform targeted strategies to improve patient outcomes.

Crucially, our study also integrates an antimicrobial stewardship perspective by providing treatment recommendations based on the observed antibiotic sensitivity of NV-HAP pathogens. This stewardship strategy is grounded in evidence-based medicine: Antimicrobial stewardship programs use coordinated interventions to optimize antibiotic use by encouraging selection of the most appropriate drug regimen [9]. Key elements include tailoring or de-escalating therapy once culture results are available to ensure effective coverage while minimizing unnecessary broad-spectrum use [10]. Indeed, international guidelines for hospital pneumonia endorse narrowing therapy based on culture and sensitivity findings as soon as possible [10]. Studies have demonstrated that implementing such evidence-based stewardship measures (e.g., adjusting antibiotics according to pathogen susceptibilities) can improve antimicrobial use without compromising clinical outcomes [9]. Aligning empiric treatment with local microbiological data is also critical for combating antimicrobial resistance (AMR) - a global health priority emphasized by organizations like the World Health Organization (WHO) and US Centers for Disease Control and Prevention (CDC) [11]. WHO identifies antibiotic stewardship programs as one of the most cost-effective strategies to optimize antibiotic use and curb resistance on a national level, and the CDC likewise stresses that hospital stewardship efforts improve patient care while fighting the threat of AMR [11,12]. By incorporating our local NV-HAP susceptibility findings into treatment recommendations, we directly support these evidence-based stewardship principles.

In summary, this retrospective observational study is the first to examine NV-HAP in Bahrain. We characterize the distribution of causative pathogens and resistance patterns of NV-HAP cases presenting to a tertiary care hospital - King Hamad University Hospital (KHUH), the largest tertiary institution in Bahrain. Our findings address a notable gap in the regional literature and aim to enhance evidence-based practice for NV-HAP, ultimately contributing to better patient outcomes and infection control in Bahrain and similar healthcare settings.

## Materials and methods

This retrospective cross-sectional study was conducted at KHUH, a tertiary care hospital in the Kingdom of Bahrain. The objective of this study is to determine the distribution of pathogens isolated from sputum cultures or bronchoalveolar lavage (BAL) samples in individuals aged 18 years and older, who were clinically diagnosed with NV-HAP and had a positive culture between January 2017 and March 2022. Furthermore, it aims to investigate the antimicrobial resistance among the identified organisms. HAP was defined as pneumonia that developed in non-intubated patients 48 hours or more after hospital admission, confirming that the infection was not incubating at the time of admission.

HAP was suspected in cases where patients exhibited one or more of the following: a temperature above 38°C without another recognized cause, leukocytosis (white blood cell count > 12,000/mm³) or leukopenia (white blood cell count < 4,000/mm³), or altered mental status in patients above 70 years without another known cause. Furthermore, at least two of the following symptoms were necessary: new or worsening purulent sputum, increased respiratory secretions or suctioning, new or worsening cough, dyspnea, tachypnea, rales or bronchial breath sounds, or worsening gas exchange (e.g., oxygen desaturation and increased oxygen demand). However, a conclusive diagnosis of HAP required radiographic evidence of a new or progressive pulmonary infiltrate.

Exclusion criteria included patients diagnosed with VAP, those receiving antimicrobial therapy before the study, infections caused by confirmed non-bacterial pathogens such as fungi or viruses, and patients enrolled in clinical trials that restricted secondary data use. Patients with compromised immune systems were also excluded, including those with leukemia, lymphoma, an absolute neutrophil count < 500/mm³, known HIV infection with a CD4 count < 200, individuals who had undergone splenectomy, recent post-transplant, were receiving cytotoxic chemotherapy, had been on high-dose steroids for more than two weeks, or were using daily immunosuppressants for longer than two weeks.

Regarding missing data, only patients with complete clinical, microbiological, and treatment data were included in the study. Records with missing key variables were excluded during the initial screening and manual chart review process. As a result, the final study cohort contained no missing data for variables included in the analysis, and no data imputation was required.

These exclusions were necessary to ensure a clinically homogeneous study population and to avoid confounding factors that could influence both the presentation and microbiological profile of pneumonia. Immunocompromised patients, for example, are predisposed to atypical pathogens, opportunistic infections, and distinct antimicrobial susceptibility patterns that differ substantially from the general NV-HAP population. Similarly, patients already on antimicrobial therapy before culture collection may have altered or suppressed bacterial growth, which would bias pathogen distribution and resistance estimates. VAP cases were excluded because they represent a separate clinical entity with different risk factors, pathogen epidemiology, and management guidelines. Excluding these groups allowed for a clearer assessment of NV-HAP caused by typical bacterial pathogens in immunocompetent adults within the hospital setting.

In KHUH, all sputum and bronchoalveolar lavage (BAL) samples submitted for suspected pneumonia during the study period were routinely cultured, ensuring that no selective culturing was performed and reducing the risk of sampling bias. Positive cultures were defined using standard quantitative thresholds: >10⁴-10⁵ CFU/mL for sputum and >10³-10⁴ CFU/mL for BAL specimens. These thresholds follow widely accepted diagnostic criteria, where lower cut-offs are used for BAL because it is a lower-respiratory specimen obtained through a more sterile sampling technique, whereas sputum requires higher thresholds due to the potential for contamination by oropharyngeal flora.

All specimens were incubated according to international clinical microbiology protocols. Culture plates were incubated at 35°C-37°C for 18-24 hours under aerobic conditions, with extended incubation up to 48 hours when initial growth was low or when slow-growing organisms were suspected. Manual colony counts were performed using a calibrated one-microliter loop to allow standardized quantification of CFUs.

Pathogen identification was performed using the Bruker MALDI-TOF mass spectrometry system, providing rapid and accurate species-level identification. Further characterization, including minimum inhibitory concentration (MIC)-based antimicrobial susceptibility testing, was completed using the BD Phoenix M50 automated system (Becton, Dickinson and Company, NJ) in accordance with standardized reference ranges. MIC testing was used to assess antibiotic resistance and guide appropriate treatment options, with multidrug resistance defined as non-susceptibility to ≥1 agent in ≥3 antimicrobial categories [13].

The ICD-10 code for HAP (U69.01) was initially used to identify potential cases from the KHUH Electronic Medical Records system. As this code does not reliably differentiate between NV-HAP and VAP, all retrieved cases underwent manual chart review. This review confirmed NV-HAP by verifying that pneumonia developed ≥48 hours after admission in non-intubated patients and ensuring that the diagnosis met clinical and radiographic criteria. This manual verification step minimized misclassification due to coding variability and ensured accurate case selection.

Ethical approval for this retrospective study was obtained from the KHUH Institutional Review Board (IRB) on June 1, 2022, prior to data extraction and analysis. All data were handled in accordance with institutional and national ethical standards for research involving patient records.

The collected data included the patient’s biographical information (gender, age, and nationality), the causative organism, and the antibiotic susceptibility profiles of the most commonly used antibiotics for treating HAP.

The antibiotics assessed in this study were those routinely included in the KHUH HAP treatment protocol and regularly tested by the microbiology laboratory for bacterial respiratory isolates. The agents evaluated were amikacin, amoxicillin-clavulanate (Augmentin), aztreonam, cefepime, cefotaxime, ceftazidime, ceftriaxone, cefuroxime, ciprofloxacin, trimethoprim-sulfamethoxazole (Cotrimoxazole), ertapenem, gentamicin, levofloxacin, meropenem, piperacillin-tazobactam, tigecycline, cefazolin, ampicillin, and sulbactam. This panel reflects the antibiotics most frequently prescribed for HAP at KHUH as well as those required for monitoring resistance trends in commonly encountered pathogens.

Although tigecycline is not a standard first-line treatment for pneumonia, it is routinely included in the laboratory’s susceptibility panel because of its relevance in detecting resistance among MDR organisms such as *A. baumannii* and other carbapenem-resistant Gram-negative bacteria. Its inclusion therefore provides important epidemiological information for understanding the local resistance landscape.

In this study, statistical analysis was performed using Statistical Package for Social Sciences (SPSS) version 25.0 (IBM Corp., Armonk, NY). Frequencies and percentages were calculated for categorical variables to provide a detailed overview. Chi-square tests were utilized to evaluate significant differences between these frequencies. A p-value of ≤0.05 was deemed to indicate statistical significance, ensuring that observed differences were not due to chance.

## Results

A total of 583 patients with a clinical diagnosis of HAP were initially screened. After applying the predefined exclusion criteria and restricting the analysis to culture-confirmed NV-HAP, 184 patients with positive bacterial cultures were included in the final analysis. This cohort, therefore, represents the distribution of isolated pathogens in culture-confirmed NV-HAP cases identified during the study period.

The final sample comprised 120 male patients (65.2%) and 64 female patients (34.8%). The distribution of comorbidities across sex and age groups is presented in Table 1. Patients were stratified into two age groups: <75 years (76 patients; 41.3%) and ≥75 years (108 patients; 58.7%). Patients aged ≥75 years demonstrated a higher distribution of comorbidities than those aged <75 years, including hypertension, cerebrovascular disease, atrial fibrillation, chronic kidney disease, chronic obstructive pulmonary disease (COPD), dementia, and epilepsy (all p < 0.01). Bronchiectasis and ischemic heart disease were more frequently observed in patients aged ≥ 75 years, although these differences were not statistically significant. In contrast, patients aged < 75 years had a higher distribution of diabetes and asthma compared with those aged ≥ 75 years (both p < 0.05).

**Table 1:** Distribution of comorbidities across genders ^a^ Percentage calculated from the male group (n = 120). ^b^ Percentage calculated from the female group (n = 64). ^c^ Percentage calculated from the total sample (n = 184). Comparisons are between male (n = 120) and female (n = 64) groups. Chi-square test was used when all expected cell counts were ≥5, with the test statistic reported as χ². Fisher’s exact test was used when any expected cell count was <5, with the test statistic reported as the odds ratio (OR).

	Male (Count (Percentage))^a^	Female (Count (Percentage))^b^	Total (Count (Percentage))^c^	Statistic (χ² or OR)	p-value
Hypertension	58 (48.3%)	46 (71.9%)	104 (56.5%)	χ² = 9.413	0.002
Stroke	51 (42.5%)	15 (23.4%)	66 (35.9%)	χ² = 6.593	0.01
Atrial fibrillation	17 (14.2%)	9 (14.1%)	26 (14.1%)	χ² = 0.000	0.985
Chronic kidney disease	49 (40.8%)	16 (25%)	65 (35.3%)	χ² = 4.580	0.032
Chronic obstructive pulmonary disease	11 (9.2%)	0 (0%)	11 (6%)	OR = ∞	0.009
Ischemic heart disease	7 (5.8%)	7 (10.9%)	14 (7.6%)	OR = 0.504	0.248
Diabetes	55 (45.8%)	28 (43.8%)	83 (45.1%)	χ² = 0.073	0.787
Bronchiectasis	9 (7.5%)	0 (0%)	9 (4.9%)	OR = ∞	0.028
Asthma	9 (7.5%)	5 (7.8%)	14 (7.6%)	OR = 0.957	1
Dementia	20 (16.7%)	0 (0%)	20 (10.9%)	χ² = 11.967	< 0.001
Epilepsy	9 (7.5%)	11 (17.2%)	20 (10.9%)	χ² = 4.043	0.044

Age stratification was performed to examine age-related differences in the distribution of isolated pathogens prior to antimicrobial resistance analyses in the overall cohort. This approach was used given the disproportionate burden of NV-HAP among elderly patients and their increased vulnerability related to frailty, aspiration risk, and comorbidity burden, with potential implications for empirical treatment and infection prevention strategies.

Twenty bacterial species were identified; however, the five most common organisms isolated were *K. pneumoniae* (38.59%), *P. aeruginosa* (26.09%), *Staphylococcus aureus* (8.70%), *Stenotrophomonas maltophilia* (6.52%), and *A. baumannii* (3.80%), with a statistically significant difference in distribution between these groups (p = 0.014). *K. pneumoniae* and *P. aeruginosa* were the most predominant species among both males and females (p < 0.001 for each organism within each group) as shown in Table 2. The frequencies of *K. pneumoniae* and *P. aeruginosa* were higher in males than in females (39.17% and 28.33% vs. 37.50% and 21.88%; p = 0.006 and 0.003, respectively). Additionally, *S. aureus* was more frequently isolated in females than in males (18.75% vs. 3.33%; p = 0.04). In addition, *K. pneumoniae* and *P. aeruginosa *were the most predominant species across both age groups (p < 0.001 for each organism within each group) as shown in Table 3. The frequency of *K. pneumoniae* was significantly lower in patients under 75 compared to those aged 75 and above (p < 0.01). No statistically significant differences were found in the frequencies of *P. aeruginosa*, *S. aureus*, *S. maltophilia*, or *A. baumannii* between the two age groups.

**Table 2:** Distribution of predominant bacterial isolates in non-ventilator hospital-acquired pneumonia stratified by gender The table presents the number and percentage of cases for the five most prevalent bacterial pathogens identified in male and female patients. ^a^ Percentages were calculated within each gender. P-values were calculated for organisms within each gender using the Chi-square test.

Bacteria	Number of cases (Percentage)^a^	Chi-square statistic	p-value
Male			
K. pneumoniae	47 (39.17%)	67.29	<0.001
P. aeruginosa	34 (28.33%)		
S. maltophilia	9 (7.50%)		
A. baumannii	5 (4.17%)		
S. aureus	4 (3.33%)		
Female			
K. pneumoniae	24 (37.50%)	26.58	<0.001
P. aeruginosa	14 (21.88%)		
S. aureus	12 (18.75%)		
S. maltophilia	3 (4.69%)		
A. baumannii	2 (3.13%)		

**Table 3:** Distribution of predominant bacterial isolates in non-ventilator hospital-acquired pneumonia stratified by age groups The table presents the number and percentage of cases for the five most prevalent bacterial pathogens identified in each age group. ^a^ Percentages were calculated within each gender. P-values were calculated for the organisms within each gender using the Chi-square test.

Bacteria	Number of cases (Percentage)^a^	Chi-square statistic	p-value
Age group 1: under 75 years			
K. pneumoniae	23 (30%)	29.76	<0.001
P. aeruginosa	20 (26%)		
S. aureus	8 (11%)		
S. maltophilia	4 (5%)		
A. baumannii	3 (4%)		
Age group 2: 75 years and older			
K. pneumoniae	48 (44%)	81.04	<0.001
P. aeruginosa	28 (26%)		
S. aureus	8 (7%)		
S. maltophilia	8 (7%)		
A. baumannii	4 (4%)		

Among the 71 *K. pneumoniae* isolates, 40 (97.56%) were MDR; of these, 22 cases (55%) were in males and 18 (45%) in females, with no statistically significant difference (p = 0.52). Additionally, 48.14% of MDR cases (13/40) were in patients under 75 years and 67.5% (27/40) in those aged 75 years and above, with a statistically significant difference (p = 0.02). Among the 48 *P. aeruginosa *isolates, seven (14.6%) were MDR. Out of these, four were males, and three were females (p = 0.371).

**Figure 1:**
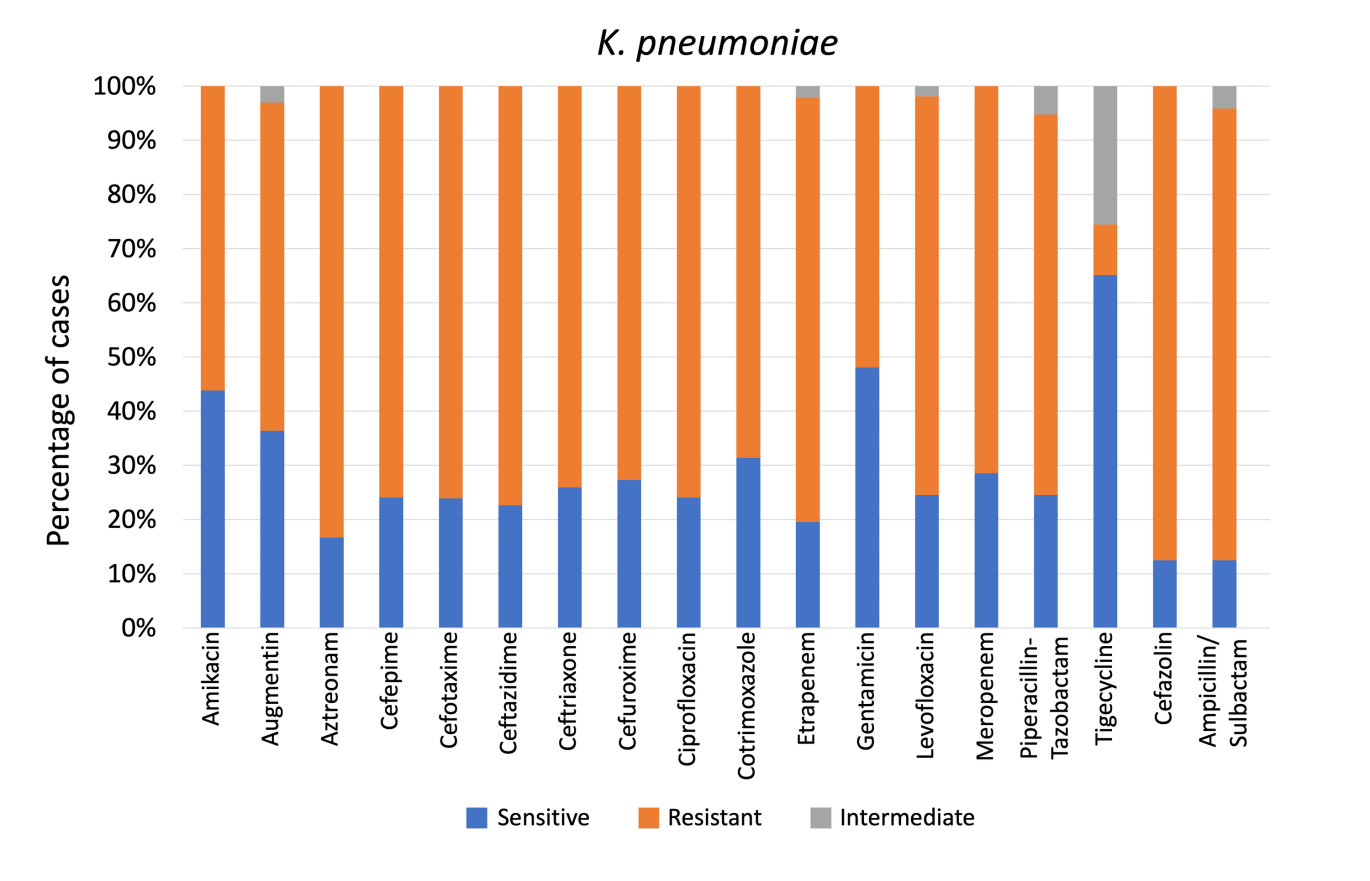
Klebsiella pneumoniae sensitivity patterns to antibiotics used to treat non-ventilator hospital-acquired pneumonia, as a percentage of cases p < 0.01 (for all mentioned antibiotics), calculated using the Chi-square test.

**Figure 2:**
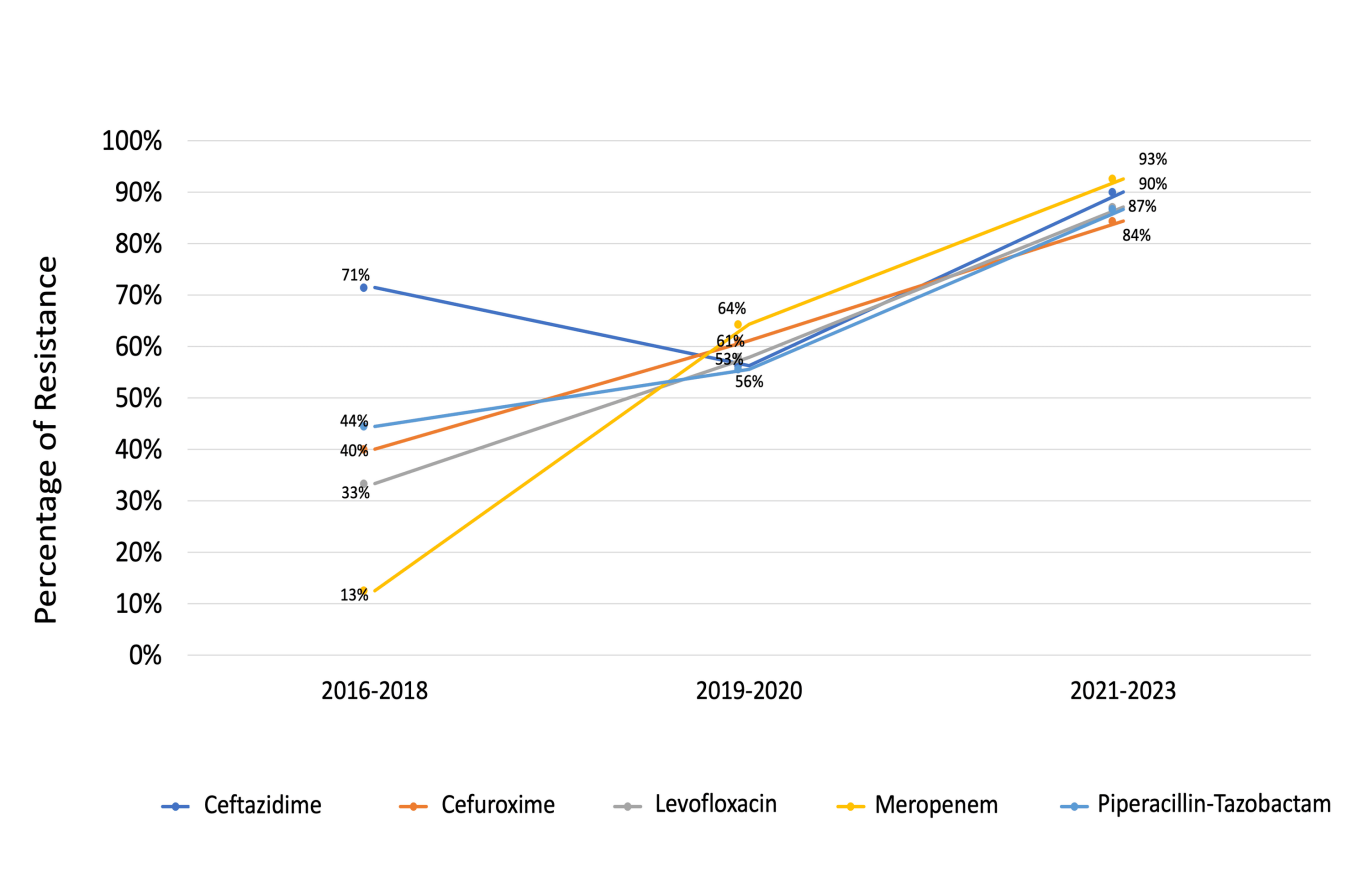
Change in antibiotic resistance of Klebsiella pneumoniae over the study period p-values (using Chi-square test) were 0.031, 0.047, 0.02, <0.01, and 0.014 for ceftazidime, cefuroxime, levofloxacin, meropenem, and piperacillin-tazobactam, respectively.

*K. pneumoniae *showed the highest resistance to cefazolin (87.5%) and then tigecycline (65.12%), followed by gentamicin (48.08%) as shown in Figure 1. Resistance of *K. pneumoniae* to ceftazidime, cefuroxime, levofloxacin, meropenem, and piperacillin-tazobactam changed significantly over the study period (p = 0.031, 0.047, 0.02, <0.01, and 0.014, respectively), as shown in Figure 2, which illustrates the temporal trends in resistance to these antibiotics.

**Figure 3:**
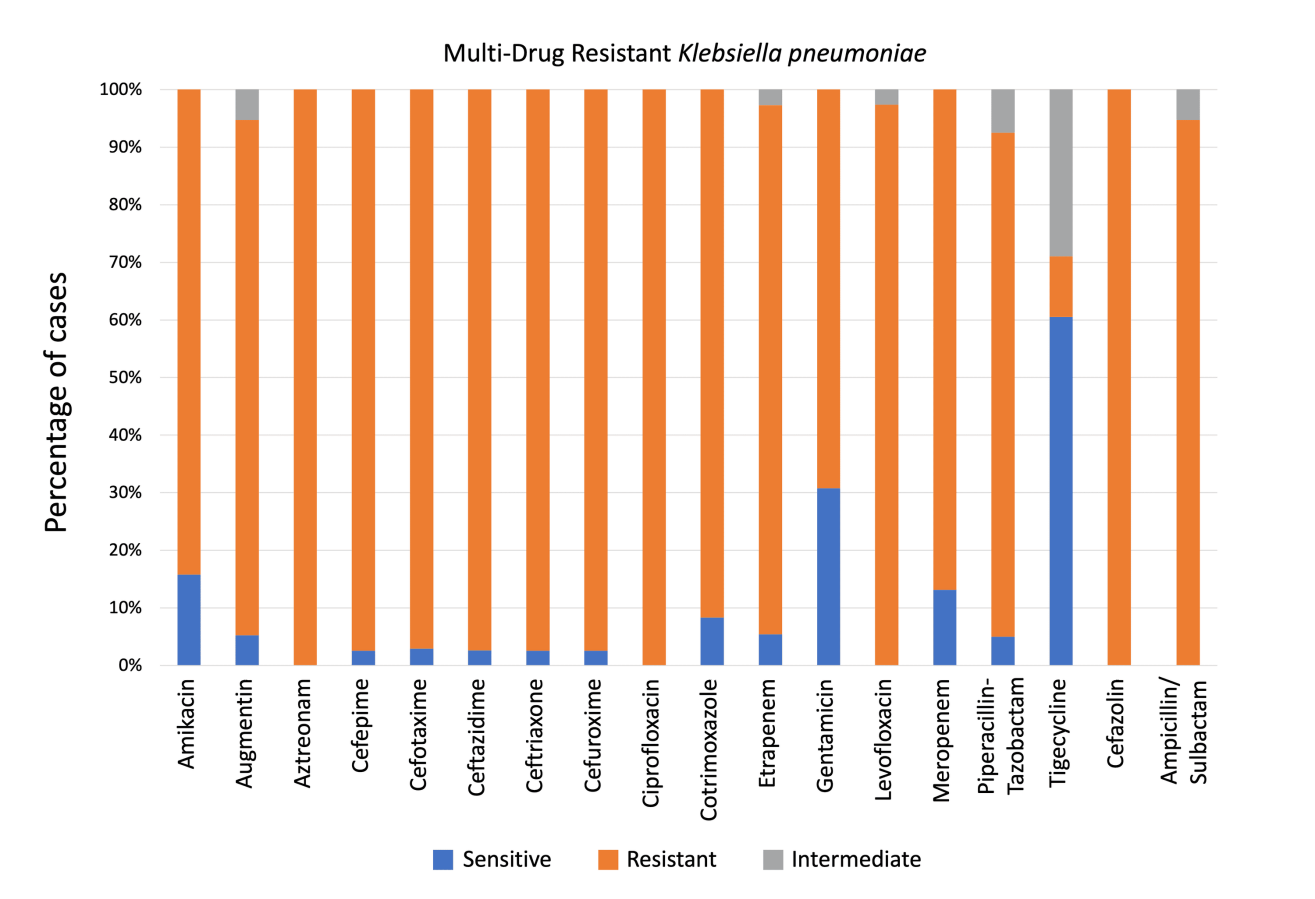
Multidrug-resistant Klebsiella pneumoniae sensitivity patterns to antibiotics used for non-ventilator hospital-acquired pneumonia, as a percentage of cases p < 0.01 (for all mentioned antibiotics) calculated using the Chi-square test.

**Figure 4:**
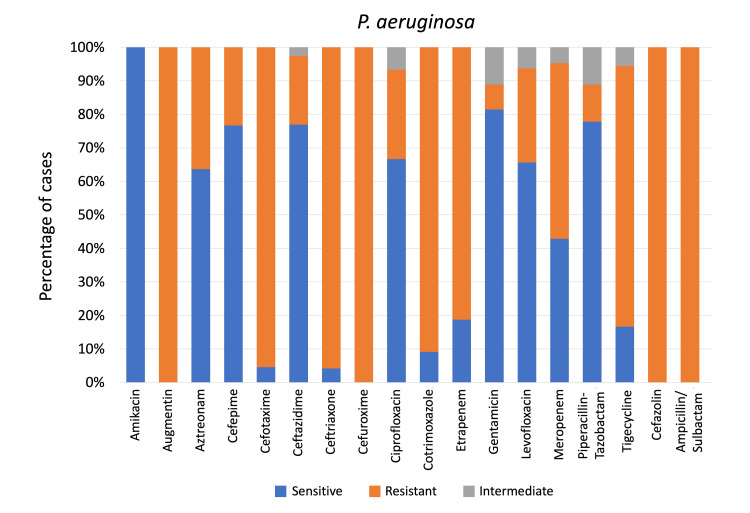
Pseudomonas aeruginosa sensitivity patterns to antibiotics used for non-ventilator hospital-acquired pneumonia, as a percentage of cases p < 0.01 (for all mentioned antibiotics) calculated using the Chi-square test.

MDR *K. pneumoniae* isolates showed 100% resistance to aztreonam, ciprofloxacin, and cefazolin, but the highest sensitivity was to tigecycline (60.52%), followed by gentamicin (30.76%) (Figure 3). *P. aeruginosa* exhibited 100% resistance to ampicillin/sulbactam, cefuroxime, augmentin, and cefazolin but showed the highest sensitivity to amikacin (100%), followed by gentamicin (81.48%) (Figure 4). MDR *P. aeruginosa* demonstrated resistance to aztreonam, cefepime, ceftazidime, ciprofloxacin, levofloxacin, piperacillin-tazobactam, with highest resistance toward meropenem (52.40%) and lowest resistance toward gentamicin (7.40%).

*S. aureus *showed 60% resistance to ciprofloxacin while displaying 100% sensitivity to aztreonam, cefepime, ceftazidime, ceftriaxone, cefuroxime, ertapenem, meropenem, piperacillin-tazobactam, tigecycline, cefazolin, and ampicillin/sulbactam. *S. maltophilia* displayed 100% resistance to 15 antibiotics, including amikacin, augmentin, aztreonam, cefepime, cefotaxime, ceftriaxone, cefuroxime, ciprofloxacin, ertapenem, gentamicin, meropenem, piperacillin-tazobactam, cefazolin, and ampicillin/sulbactam. It showed 100% sensitivity to only two antibiotics: co-trimoxazole and tigecycline. Table 4 presents the resistance rates of the most predominant organisms to commonly used antibiotics, as well as overall resistance rates regardless of organism. It also includes the number of cases in which resistance testing for a specific antibiotic was not performed.

**Table 4:** Antibiotic resistance rates in the most predominant organisms causing non-ventilator hospital-acquired pneumonia in Bahrain ^a^ Resistance percentages were calculated as the number of resistant cases/total number of cases (excluding the number of cases in which the test was not done), followed by the exact values used to calculate the percentage using the mentioned formula. ^b^ “Not tested” represents the number of cases in which the resistance of this specific antibiotic was not tested. MDR: Multidrug resistant.

	*K. pneumoniae *(n = 71)		MDR *K. pneumonia*e (n = 40)		*P. aeruginosa *(n = 48)		*S. aureus* (n = 16)		*S. maltophillia *(n = 12)	
	Resistance %^a^	Not tested^b^	Resistance %^a^	Not tested	Resistance %^a^	Not tested^b^	Resistance %^a^	Not tested^b^	Resistance %^a^	Not tested^b^
Amikacin	56.1% (32/57)	14	84.2% (32/38)	2	0% (0/32)	16	50% (1/2)	14	100% (6/6)	6
Augmentin	60.6% (20/33)	38	89.5% (17/19)	21	100% (11/11)	37	23.08% (3/13)	3	100% (3/3)	9
Aztreonam	83.3% (20/24)	47	100% (20/20)	20	36.36% (4/11)	37	0% (0/1)	15	100% (1/1)	11
Cefepime	75.9% (41/54)	17	97.4% (38/39)	1	23.33% (7/30)	18	0% (0/11)	5	100% (4/4)	8
Cefotaxime	76.1% (35/46)	25	97.1% (33/34)	6	95.45% (21/22)	26	13.33% (2/15)	1	100% (6/6)	6
Ceftazidime	75.9% (41/54)	17	97.4% (37/38)	2	20.51% (8/39)	9	0% (0/4)	12	66.67% (6/9)	3
Ceftriaxone	74.1% (40/54)	17	97.4% (38/39)	1	95.83% (23/24)	24	0% (0/12)	4	100% (6/6)	6
Cefuroxime	72.7% (40/55)	16	97.4% (38/39)	1	100% (21/21)	27	0% (0/12)	4	100% (7/7)	5
Ciprofloxacin	75.9% (41/54)	17	100% (39/39)	1	26.67% (8/30)	18	60% (9/15)	1	100% (1/1)	11
Cotrimoxazole	68.6% (35/51)	20	91.7% (33/36)	4	90.91% (20/22)	26	23.08% (3/13)	3	0% (0/11)	1
Ertapenem	78.3% (36/46)	25	91.9% (34/37)	3	81.25% (13/16)	32	0% (0/1)	15	100% (6/6)	6
Gentamicin	51.9% (27/52)	19	69.2% (27/39)	1	7.41% (2/27)	21	7.14% (1/14)	2	100% (5/5)	7
Levofloxacin	73.6% (39/53)	18	97.4% (37/38)	2	28.13% (9/32)	16	44.44% (4/9)	7	22.22% (2/9)	3
Meropenem	71.4% (35/49)	22	86.8% (33/38)	2	52.38% (11/21)	27	0% (0/1)	15	100% (6/6)	6
Piperacillin-Tazobactam	70.2% (40/57)	14	87.5% (35/40)	0	11.11% (4/36)	12	0% (0/9)	7	100% (5/5)	7
Tigecycline	9.3% (4/43)	28	10.5% (4/38)	2	77.78% (14/18)	30	0% (0/3)	13	N/A	12
Cefazolin	87.5% (7/8)	63	100% (7/7)	33	100% (4/4)	44	0% (0/2)	14	100% (2/2)	10
Ampicillin/Sulbactam	83.3% (20/24)	47	94.7% (18/19)	21	100% (11/11)	37	0% (0/1)	15	100% (3/3)	9

## Discussion

Distribution of isolated pathogens in NV-HAP

In our study of culture-confirmed NV-HAP in Bahrain, 120 of 184 cases (65.2%) occurred in males and 64 (34.7%) in females, yielding a male-to-female ratio of 1.88:1. This significant male predominance (p < 0.001) is consistent with findings from Al-Ghizawi et al. (1.22:1) [14]. Male predominance may be partly explained by a higher burden of comorbidities among male patients, as shown in Table 1 of this study [14]. Both studies demonstrate a clear male predominance in pneumonia, further supported by reports of male predominance in VAP and [14,15]. Additionally, an Indian ICU series by Patel et al. reported that 83.3% of NV-HAP patients were male [16].

In our study, males had a higher burden of comorbidities. In our cohort, men also carried more comorbidities. Notably, COPD was significantly more common in males (9.2%) than females, as shown in Table 1, aligning with findings of increased tobacco use among males across pneumonia types (HAP, NV-HAP, and VAP), which may contribute to higher pneumonia case representation [17-19]. Smoking, more common in males, raises risks for respiratory diseases like COPD, further supporting the gender disparity in pneumonia [18].

For example, Fortaleza et al. showed that older age, neurologic impairment (e.g., stroke or altered consciousness), and antacid use were independent predictors of NV-HAP [20]. Hormonal differences also play a role, as androgens can reduce male immunity, while estrogens enhance immune responses in females, potentially explaining the higher proportion of NV-HAP cases observed in men [21,22]. Together, these findings suggest that gender-related factors, including a higher burden of comorbidities among men, may contribute to increased NV-HAP risk. *K. pneumoniae* (38.59%) and *P. aeruginosa* (26.09%) were the most common pathogens associated with hospital‑acquired pneumonia in this non‑ventilated adult cohort, a distribution consistent with the microbiological profile reported in NV‑HAP and non‑ventilated HAP literature [23-26]. Large epidemiological studies have demonstrated that NV‑HAP is associated with substantial morbidity and mortality, including prolonged length of stay and increased in‑hospital mortality, underscoring the clinical relevance of pathogen distribution in this population [23,27]. The predominance of *K. pneumoniae* observed in the present study aligns with multicountry Asian surveillance data reported by Chung et al., where *K. pneumoniae* was the leading HAP pathogen in several countries, and *P. aeruginosa* consistently ranked among the most common organisms, including in non‑ventilated patients [25]. The significantly higher frequency of* K. pneumoniae* among patients aged ≥75 years may be explained by age‑related factors frequently described in NV‑HAP literature, including immunosenescence, increased aspiration risk due to frailty and dysphagia, and greater cumulative healthcare and antibiotic exposure, which favor colonization and infection with Enterobacterales [24,28,29]. While studies from the Gulf Cooperation Council (GCC) region commonly report hospital‑acquired and ventilator‑associated pneumonia together rather than isolating NV‑HAP cohorts, regional data consistently identify *K. pneumoniae* and *P. aeruginosa* as dominant hospital‑acquired respiratory pathogens, supporting the external validity of these findings [5,30]. However, Bahrain‑specific adult NV‑HAP microbiological data remain absent from the indexed peer‑reviewed literature, with available local studies focusing primarily on ventilator‑associated pneumonia, highlighting a significant regional evidence gap that the present study helps to address [7].

Available data on hospital-acquired pneumonia in the Middle East largely come from VAP studies, with little focus on NV-HAP in Bahrain or neighboring countries. In lieu of regional NV-HAP data, we compared our findings with these VAP studies. Notably, a 10-year Lebanese ICU study reported Gram-negatives predominating in VAP (MDR *A. baumannii *~ 33% of isolates, followed by *P. aeruginosa* ~17%, and *E. coli* ~12%) [6]. Likewise, a Saudi analysis found *A. baumannii *to be the most frequent pathogen in late-onset VAP [31]. In the UAE, a 22-month prospective study of VAP in a tertiary hospital observed *K. pneumoniae* as the leading organism (21% of isolates), followed by *S. aureus *and *P. aeruginosa* (16% each) [32]. By contrast, our Bahrain NV-HAP cases were dominated by *K. pneumoniae* (38.6% of isolates) and *P. aeruginosa* (26.1%), with *A. baumannii *(only 3.8%). This suggests a marked difference in pathogen distribution between VAP and NV-HAP in the region. Indeed, *K. pneumoniae* was more disproportionately common in our NV-HAP cohort than in regional VAP studies (e.g., 38.6% vs 21% in UAE), whereas *A. baumannii *- a mainstay of ICU ventilated infections - was relatively uncommon [32]. These findings align with global observations that etiologic profiles differ between ventilated and non-ventilated pneumonia patients: For example, a large Chinese study found that non-VAP HAP cases had significantly more *Klebsiella *(and other *Enterobacteriaceae*) and fewer *Acinetobacter *compared to VAP cases [33]. Our data confirm this trend in a Middle Eastern context, underscoring that NV-HAP may have a distinct microbial pattern even within a region historically emphasizing VAP data. Importantly, we could not identify prior NV-HAP-specific studies from Bahrain or the Gulf, highlighting the novelty of our report in filling this regional knowledge gap.

In our NV-HAP isolates, other notable pathogens (in descending order of frequency) were *S. aureus* (8.7% of isolates), *S. maltophilia* (6.3%), and *A. baumannii* (3.8%). *S. aureus* (often methicillin-resistant *S. aureus*, MRSA) is a well-recognized cause of HAP globally, though its frequency in our NV-HAP cohort was relatively low [34,35]. For instance, *S. aureus *accounted for 16% of VAP isolates in the UAE study and about 12.6% of HAP pathogens in a large Chinese study, considerably higher than Bahrain’s 8.7%. An even higher rate (~25%) was seen in one Egyptian hospital HAP/VAP cohort, likely reflecting local ICU outbreaks of MRSA [32,33,36]. The modest contribution of *S. aureus* in Bahrain’s NV-HAP might be due to differences in patient population or infection control success with MRSA, but it remains an important consideration given its virulence. *S. maltophilia*, an intrinsically drug-resistant environmental bacterium, comprised 6.3% of our NV-HAP isolates. This organism is an emerging opportunistic pathogen in hospital settings, especially among patients with prior broad-spectrum antibiotic exposure or comorbidities [5]. Reports of *S. maltophilia* causing HAP are relatively infrequent; for example, an Egyptian nosocomial pneumonia study found it in only ~2% of cases, yet our higher rate suggests that *S. maltophilia *may be a noteworthy NV-HAP pathogen in Bahrain [36]. Its presence underscores the need for diligent antibiotic stewardship, as selection pressure can favor such intrinsically MDR flora. Finally, *A. baumannii *was strikingly low in our NV-HAP series (3.8%), especially when compared to its prominence in regional VAP reports [6,31]. *A. baumannii* is classically associated with ventilated ICU patients and long hospital stays; our low NV-HAP rate likely reflects the fact that many NV-HAP cases occur in general wards or earlier during hospitalization, with fewer of the risk factors that facilitate *Acinetobacter* infection. Consistently, studies have noted that *Acinetobacter *prevalence drops outside the ventilated ICU setting [33]. Thus, the pathogen profile of Bahrain’s NV-HAP skews more toward enteric Gram-negatives (*Klebsiella *and *E. coli*) and *Pseudomonas*, with less involvement of the highly drug-resistant non-fermenters like *Acinetobacter *or even *Stenotrophomonas *than seen in ICU-focused studies. This difference has practical implications: Empiric therapy for NV-HAP on the wards in our hospital may not require the same extensive MDR coverage (e.g., for *Acinetobacter*) that an ICU VAP would, though coverage for *Klebsiella/Pseudomonas* and MRSA should still be considered based on our data and other HAP guidelines [34,35]. We also note that *S. pneumoniae *- a frequent cause of community-acquired pneumonia - was not among the top NV-HAP pathogens in our study, likely due to our focus on hospital-onset cases and possibly effective pneumococcal vaccination in adults; this contrasts with non-hospital settings where *S. pneumoniae* remains common. Overall, our NV-HAP pathogen spectrum is consistent with a growing body of evidence that non-ICU HAP has a distinct microbiological makeup, sharing some overlap with VAP but with important differences in the rank order of organisms.

The geographical variation in NV-HAP pathogen distribution is complex and influenced by multiple factors [5]. Our findings from Bahrain, particularly the exceptionally high prevalence of* K. pneumoniae*, may reflect unique regional and institutional factors. *K. pneumoniae* is a ubiquitous nosocomial pathogen worldwide, but the magnitude seen here is unusual: Globally, this organism accounts for roughly 11%-12% of HAP and only ~7% of non-ventilator pneumonia cases, whereas in our Bahrain NV-HAP, it constituted 38.6% [37]. This disparity suggests local ecological conditions that favor *Klebsiella’s* spread, such as hospital environment reservoirs (e.g., contaminated surfaces or equipment) and climatic factors that might facilitate Gram-negative survival. Bahrain’s high *Klebsiella *rate might also be linked to its diverse patient population. The country’s population is ~53% expatriates (largely from Asia, the Middle East, and Africa), meaning the hospital serves individuals from varied backgrounds who may carry different microbial flora or resistance genes [38]. Continuous population movement and cross-border patient care in the Gulf have been implicated in the introduction and dissemination of resistant strains; thus, Bahrain’s role as a regional hub could contribute to a broader mix of hospital pathogens [5].

Patient demographics and comorbidities in our cohort (all adults ≥ 18, with many likely elderly or chronically ill) further influence susceptibility - for instance, diabetic or immunosuppressed patients are known to be more prone to Gram-negative pneumonias [37]. Clinical practice patterns are another consideration: Heavy empirical use of broad-spectrum antibiotics can be selected for organisms like *Klebsiella *and *Stenotrophomonas*, whereas stringent infection control can limit spread of others like *Acinetobacter*. Differences in healthcare infrastructure and guidelines adherence between regions may partly explain why MRSA is a dominant HAP pathogen in some Western settings, while Gram-negatives prevail in many Middle Eastern hospitals [5,34,35]. In sum, Bahrain’s NV-HAP microbiology appears to be shaped by a confluence of regional factors - environmental, demographic, and systemic. This underlines the importance of continuous local surveillance and regional comparisons.

Antibiotic resistance patterns

Our study, in line with findings from Iraq, Thailand, and other parts of Asia and the Middle East, shows significant resistance of *K. pneumoniae* to beta-lactam antibiotics, particularly penicillins and cephalosporins [25,39,40]. Resistance to third-generation cephalosporins was also high in our data: cefazolin 87.5%, ceftriaxone 74.07%, and cefotaxime 76.09%, comparable to Iraq (76.09%) [41]. Other studies in Thailand and Asia reported extended spectrum beta-lactamase (ESBL)-producing isolates at 41.4% and 47%, respectively [25,39], which are often resistant to these antibiotics. ESBL production was not tested in our study but likely contributes to the observed resistance. In the Middle East, ceftazidime resistance ranged from 74.6% to 92.5% in Saudi Arabia, supporting a broader regional trend that includes Bahrain [42].

High carbapenem resistance was noted in our KHUH data, with *K. pneumoniae* showing 78.3% resistance to ertapenem and 71.4% to meropenem. Moreover, over the 2016-2022 study period, *K. pneumoniae* meropenem resistance increased significantly from 13% to 93% (p < 0.01). In contrast, other regions reported much lower carbapenem resistance; for example, a Thai hospital observed that ~99% of *K. pneumoniae* isolates remained carbapenem-sensitive, and a multicenter Asian study found only about 2.2% resistance to imipenem [25,39]. Similarly, US surveillance (2009-2012) indicated >90% susceptibility to meropenem in *K. pneumoniae* from pneumonia patients [43]. Globally, rising trends in *K. pneumoniae* carbapenem resistance have been well documented. In China, the prevalence of carbapenem-resistant *K. pneumoniae* in HAP climbed from 0.8% in 2007 to 11.6% in 2016 [33]. A decade-long Korean study likewise reported *K. pneumoniae* carbapenem non-susceptibility increasing from 0% (around 2011) to 38% by 2021 [44]. In Saudi Arabia, resistance to meropenem and imipenem has reached 95% and 96.3%, largely due to carbapenemase-producing strains like OXA-48 and NDM-1 [42,45]. Indeed, a recent Bahraini study detected bla NDM-1 and bla OXA types in over 90% of clinical carbapenem-resistant *K. pneumoniae* isolates, underscoring the regional dissemination of these carbapenemases [46]. These trends highlight the need for ongoing surveillance (including molecular monitoring for carbapenemase genes), strict antibiotic stewardship, and exploration of alternative therapies to combat the rising carbapenem resistance.

High fluoroquinolone resistance was observed, with rates of 75.93% for ciprofloxacin and 73.58% for levofloxacin, indicating reduced effectiveness against *K. pneumoniae* in NV- HAP. Notably, levofloxacin resistance increased from 33% in 2016 to 87% in 2022 during our study period (p = 0.02), underscoring a significant upward trend. Similar resistance rates were reported in Iraq (75.93% for ciprofloxacin) and Egypt (83.3%), and a Saudi Arabian hospital observed fluoroquinolone resistance exceeding 70% by the late 2010s, while lower rates were found in Asia (31.2%) and the United States (only 75.7% sensitivity to levofloxacin) [25,39,40,41,43,47,48].

Bahrain’s escalating rates appear to be regionally significant, suggesting fluoroquinolones may no longer be reliable for treating NV-HAP and highlighting an urgent need for alternative strategies. This concern aligns with broader trends, as global surveillance indicates that fluoroquinolone effectiveness against *K. pneumoniae* is steadily declining [49]. These findings reinforce the need for immediate action and alternative therapies to manage NV-HAP in the face of rising resistance.

Tigecycline was the most effective antibiotic against *K. pneumoniae*, with a 65.12% sensitivity rate, aligning with higher rates reported in studies from the United States (97.3%) and the Eastern Mediterranean region (99.3%). Tigecycline resistance remained low (9.3%), consistent with findings from Saudi Arabia (15.7%), indicating that it remains a reliable option for treating NV-HAP [50]. Amikacin resistance was 56.14%, similar to rates found in Iran (56.52%) but lower than those in Egypt (61.1%) [40,51]. Resistance rates for gentamicin (51.92%) and amikacin (56.14%) were also consistent with findings from Saudi Arabia, suggesting that while aminoglycosides retain some efficacy, resistance levels warrant caution [50]. Despite efficacy, tigecycline’s use is limited by poor lung penetration.

A key concern is the high prevalence of MDR *K. pneumoniae* strains, with 97.56% of isolates in our study classified as MDR. This is significantly higher than the 44.7% MDR rate reported for *K. pneumoniae* in Asia [25]. Thailand highlighted the role of ESBL-producing *K. pneumoniae* in contributing to MDR through resistance across multiple antibiotic classes [39]. Al-Baz et al. reported that 97.2% of carbapenem-resistant *K. pneumoniae *isolates were extensively drug-resistant (XDR), while Khairy et al. found 100% MDR among *K. pneumoniae* isolates in Egypt [40,48]. This widespread MDR limits treatment options and poses a significant public health issue.

Overall, this study, conducted in a tertiary care center in Bahrain, found increasing resistance of *K. pneumoniae* in NV-HAP to ceftazidime, cefuroxime, levofloxacin, meropenem, and piperacillin-tazobactam. These trends were specific to our local setting and indicate a growing challenge in managing *K. pneumoniae* infections. Compared to lower resistance rates reported in Asia, our findings suggest a more serious regional concern [25]. This trend also parallels observations from the United States, where resistance patterns continue to evolve, highlighting the need for robust antimicrobial stewardship and infection prevention strategies [43].

The rising prevalence of *K. pneumoniae*, especially carbapenem-resistant strains, is a growing global concern. Choi et al. observed an increase in *K. pneumoniae* as a cause of HAP alongside a decline in *S. aureus* cases from 2011 to 2021, indicating a shift in dominant pathogens [44]. The COVID-19 pandemic - overlapping with our study period - and higher *K. pneumoniae* prevalence in patients aged ≥75 years have contributed to this trend, with infections surging compared to pre-pandemic levels [44]. Likely drivers of this increase include the expanded use of invasive devices, prolonged hospital stays, and heightened antibiotic use [44]. Research underscores that Gram-negative bacteria, especially *K. pneumoniae*, now play a critical role in hospital-associated infections [52].

A comparison with a study on carbapenem-resistant *K. pneumoniae* (CRKP) infections in Eastern China clearly highlights both similarities and differences in resistance trends [53]. While their study specifically investigated CRKP, our findings - though not CRKP-confirmed - showed substantial resistance that raises concern. In our Bahrain-based cohort, ertapenem resistance was 78.26%, and meropenem resistance was 71.43%, suggesting the possible presence of CRKP strains. Both studies reported high resistance to beta-lactam antibiotics, with complete resistance to ampicillin and significant resistance to cefazolin (87.5%), ceftriaxone (74.07%), and ceftazidime (77.36%) in our data, aligning with the Chinese study’s findings of 100% resistance to cefazolin and ceftriaxone. Resistance to combination therapies was also notable: ampicillin/sulbactam (83.33%) and piperacillin/tazobactam (70.18%) in our study, versus 100% and 97.3%, respectively, in the Chinese cohort. Shared resistance mechanisms may underlie these trends. Although CRKP was not specifically tested in our study, the high levels of carbapenem resistance are suggestive and warrant further confirmatory testing for carbapenemase-producing strains.

*P. aeruginosa* is intrinsically resistant to several antibiotics, including aminopenicillins, early-generation cephalosporins, ceftriaxone, cefotaxime, and ertapenem [54]. In our Bahrain cohort, this was reflected by near-complete resistance to ceftriaxone and cefotaxime (~95%), underscoring their ineffectiveness for pseudomonal pneumonia. Such high resistance contrasts with lower rates in Western settings, where these agents are rarely used or tested against *P. aeruginosa* [43]. Accordingly, we focused on antipseudomonal β-lactams such as piperacillin-tazobactam, ceftazidime, cefepime, and aztreonam.

Encouragingly, our isolates showed better susceptibility profiles compared to regional and global data. Piperacillin-tazobactam resistance was only 11%, and susceptibility to ceftazidime and cefepime was ~77%, which is significantly better than resistance rates reported in large Asian studies (~35%-37%) and in Saudi Arabia (~40%-50%) [45,55]. Thai data also showed lower susceptibilities (72.4% for piperacillin-tazobactam and 57.6% for ceftazidime) [39]. When compared to the United States and Europe, where susceptibility to piperacillin-tazobactam is ~63-73%, our rates were more favorable [43]. Aztreonam showed moderate activity (63.6%), aligning with Saudi data [45]. However, in MDR cases elsewhere (e.g., Germany), resistance to these β-lactams can exceed 50%-60% [56].

Aminoglycosides demonstrated excellent efficacy. All isolates were susceptible to amikacin (100%), and gentamicin was effective in ~81% of cases. Similar findings are reported globally: Thai hospitals reported 95% susceptibility to netilmicin, and studies from Saudi Arabia and Germany also confirm high aminoglycoside activity (e.g., <10% resistance to amikacin) [39,45,56]. Even among MDR strains, aminoglycosides remain effective - German data show only 21% gentamicin resistance and 27% to tobramycin [56]. Our findings reinforce amikacin’s continued clinical value in HAP/NV-HAP.

Carbapenem resistance, however, remains a major concern. In our study, only ~43% of isolates were susceptible to meropenem, and 52.4% were resistant. Ertapenem was ineffective (81% resistant), consistent with its intrinsic resistance profile [54]. These rates mirror regional data, with over 50% carbapenem resistance in Saudi hospitals [45] and similarly high rates reported in China and Germany [55,56]. Even historical surveillance (SENTRY 2009-2012) showed ~34% non-susceptibility in Europe [43]. Rising carbapenem resistance - driven by carbapenemase genes and efflux/porin mutations - threatens treatment efficacy and highlights the need for newer agents like ceftazidime-avibactam or cefiderocol [57].

Fluoroquinolone susceptibility was moderate: 26.7% resistance to ciprofloxacin and 28.1% to levofloxacin. This aligns with Asian data (~30% resistance) and Saudi reports (~67.5% susceptibility) [45,55]. Western data show slightly better rates (e.g., ~75% levofloxacin susceptibility in Germany) [56], though severe declines have been noted in certain centers (e.g., 10% susceptibility in Thai VAP cases) [39]. Given that fluoroquinolones are one of the few oral treatment options for *P. aeruginosa*, prudent use is essential to preserve their utility.

Overall, *P. aeruginosa* remains a challenging NV-HAP pathogen due to its multidrug resistance. While Bahrain showed relatively favorable susceptibility to key antipseudomonal agents, the high resistance to carbapenems and ceftriaxone underscores the importance of local resistance surveillance and appropriate empiric therapy. The consistent efficacy of aminoglycosides is encouraging [39,56], but stewardship efforts are vital to curb rising resistance and optimize patient outcomes. Globally and locally, *P. aeruginosa* demands continued vigilance and targeted antimicrobial strategies [57].

This study found 26.67% resistance to ciprofloxacin and 28.13% to levofloxacin among *P. aeruginosa* isolates. Asian studies reported similar ciprofloxacin resistance (around 30%), while Thailand experienced a sharp decline in ciprofloxacin sensitivity, down to approximately 10% [25,39]. Our findings indicate a moderate level of resistance in Bahrain, which is locally significant and underscores the need for continued surveillance and antimicrobial stewardship. These resistance rates are lower than those reported in Germany (61.5% for ciprofloxacin) [58], comparable to Saudi Arabia (67.5% sensitivity for ciprofloxacin), while US levofloxacin sensitivity remains higher at 70.5% [45].

In our HAP study, *S. aureus* showed 100% resistance to ampicillin, consistent with findings from Saudi Arabia (41.3% susceptibility) [59]. Ciprofloxacin resistance was significant at 60%, aligning with findings from Asia (78.2%), indicating limited fluoroquinolone efficacy [25]. We observed 100% sensitivity to broad-spectrum antibiotics like aztreonam and meropenem, similar to results from Thailand with vancomycin, suggesting these as effective treatment options [39]. Our data also reflect trends observed in Korea, with rising resistance and a decline in methicillin resistance [44].

For *A. baumannii* in NV-HAP, we observed 100% resistance to multiple antibiotics, including amikacin, aztreonam, and meropenem, paralleling high resistance rates reported in Asia (e.g., 67.3% for imipenem) [25]. Our findings of significant multidrug resistance align with broader findings in Asia (87% resistance) and Thailand, where declining cefoperazone/sulbactam sensitivity was noted [39,58]. Ultimately, the high resistance rates in our study highlight a concerning trend consistent with literature previous literature, particularly *A. baumannii*, which emphasize the need for continued surveillance and effective antimicrobial stewardship.

This study has certain limitations, despite the fact that it offers useful information on the frequency and resistance trends of NV-HAP pathogens in Bahrain. The retrospective nature of the study may introduce inherent biases, particularly in the selection of patients and the availability of clinical data. The results may not be generalizable to patients with prior antimicrobial exposure or immunocompromised conditions, as these groups were excluded from the analysis. Furthermore, because the study was limited to a single tertiary care institution, it is possible that the findings do not accurately reflect wider epidemiological patterns in Bahrain or the Gulf. The small sample sizes for some pathogens, e.g., *S. aureus* (n = 16), *S. maltophilia* (n = 12), and *A. baumannii* (n = 7), may also limit statistical power, which was not accounted for in the current analysis. Additionally, clinical outcomes were not assessed, limiting the ability to correlate resistance patterns with prognosis or treatment success. The study also highlights a broader limitation in the regional literature, as there remains a significant data gap on non-ventilator HAP in Bahrain and the Gulf region. Despite these limitations, this study provides valuable baseline data on the epidemiology and resistance patterns of NV-HAP in Bahrain. Future research should aim to incorporate advanced molecular diagnostics, expand the scope of antibiotic resistance testing, and explore the genetic mechanisms driving resistance in key pathogens. By addressing these gaps, future studies could further elucidate the complex interplay between hospital-acquired infections, antimicrobial resistance, and patient outcomes, ultimately leading to improved strategies for managing NV-HAP in the region.

## Conclusions

In this first Bahrain-based study of NV-HAP, *K. pneumoniae* and *P. aeruginosa* were the most frequently isolated pathogens, with a notably higher frequency of *K. pneumoniae* among patients aged ≥75 years. High resistance rates, particularly to third-generation cephalosporins, carbapenems, and fluoroquinolones, underscore the need to revise empiric therapy and strengthen antimicrobial stewardship. Compared to regional VAP data, our findings confirm that NV-HAP has a distinct microbial distribution, with lower frequencies of *A. baumannii* and *S. aureus*. This study addresses a significant regional data gap and provides essential baseline evidence to inform future infection control and antibiotic strategies in Bahrain and the Gulf.
